# Mobile Health Interventions for Self-Control of Unhealthy Alcohol Use: Systematic Review

**DOI:** 10.2196/10899

**Published:** 2019-01-29

**Authors:** Ting Song, Siyu Qian, Ping Yu

**Affiliations:** 1 Centre for IT-Enabled Transformation, School of Computing and Information Technology Faculty of Engineering and Information Sciences University of Wollongong Wollongong Australia; 2 Illawarra Health and Medical Research Institute University of Wollongong Wollongong Australia

**Keywords:** systematic review, alcohol drinking, self-control, mobile health, mHealth, treatment outcome

## Abstract

**Background:**

Unhealthy alcohol use (UAU) is one of the major causes of preventable morbidity, mortality, and associated behavioral risks worldwide. Although mobile health (mHealth) interventions can provide consumers with an effective means for self-control of UAU in a timely, ubiquitous, and cost-effective manner, to date, there is a lack of understanding about different health outcomes brought by such interventions. The core components of these interventions are also unclear.

**Objective:**

This study aimed to systematically review and synthesize the research evidence about the efficacy of mHealth interventions on various health outcomes for consumer self-control of UAU and to identify the core components to achieve these outcomes.

**Methods:**

We systematically searched 7 electronic interdisciplinary databases: Scopus, PubMed, PubMed Central, CINAHL Plus with full text, MEDLINE with full text, PsycINFO, and PsycARTICLES. Search terms and Medical Subject Headings “mHealth,” “text message,” “SMS,” “App,” “IVR,” “self-control,” “self-regulation,” “alcohol*,” and “intervention” were used individually or in combination to identify peer-reviewed publications in English from 2008 to 2017. We screened titles and abstracts and assessed full-text papers as per inclusion and exclusion criteria. Data were extracted from the included papers according to the Consolidated Standards of Reporting Trials-EHEALTH checklist (V 1.6.1) by 2 authors independently. Data quality was assessed by the Mixed Methods Appraisal Tool. Data synthesis and analyses were conducted following the procedures for qualitative content analysis. Statistical testing was also conducted to test differences among groups of studies.

**Results:**

In total, 19 studies were included in the review. Of these 19 studies, 12 (63%) mHealth interventions brought significant positive outcomes in improving participants’ health as measured by behavioral (n=11), physiological (n=1), and cognitive indicators (n=1). No significant health outcome was reported in 6 studies (6/19, 32%). Surprisingly, a significant negative outcome was reported for the male participants in the intervention arm in 1 study (1/19, 5%), but no change was found for the female participants. In total, 5 core components reported in the mHealth interventions for consumer self-control of UAU were context, theoretical base, delivery mode, content, and implementation procedure. However, sound evidence is yet to be generated about the role of each component for mHealth success. The health outcomes were similar regardless of types of UAU, deployment setting, with or without nonmobile cointervention, and with or without theory.

**Conclusions:**

Most studies reported mHealth interventions for self-control of UAU appeared to be improving behavior, especially the ones delivered by short message service and interactive voice response systems. Further studies are needed to gather sound evidence about the effects of mHealth interventions on improving physiological and cognitive outcomes as well as the optimal design of these interventions, their implementation, and effects in supporting self-control of UAU.

## Introduction

### Background

Unhealthy alcohol use (UAU) is one of the major causes of preventable morbidity, mortality, and related behavioral risks around the world [1,2]. Approximately 3.3 million deaths, accounting for 5.9% of global deaths, were caused by alcohol-related problems annually [3]. Nearly 81% of adults in Australia and 70% in Europe consume alcohol [3,4]. UAU contributed to around 70,000 Australian emergency department presentations in 2014 and 2015 and 77,000 Canadian hospitalizations in 2015 and 2016 [5,6]. It might cause allergic reactions, hormonal disturbances, and intoxication [7,8]. Over time, it might cause diseases such as alcoholic hepatitis, diabetes, cardiovascular and cerebrovascular diseases [9], or psychological problems such as depression, obsession, mania, and suicide [10,11]. Once the brain and neurons are anesthetized, a person might lose self-control [12], leading to social problems such as conflicts, unprepared sexual activities, drunk driving, and violence [13,14]. Therefore, UAU is not only a profound public health challenge but also a social concern.

As an umbrella term, UAU covers various degrees of negative effects of alcohol use on people’s well-being [15]. According to the severity, there are 2 major types of UAU: risky drinking and alcohol use disorder (AUD) [15,16].

Risky drinking is also known as problematic drinking, harmful alcohol use, risky single-occasion drinking (RSOD), or heavy episodic drinking. It refers to alcohol use that leads to the risk of negative health consequences [16]. It can be measured by the number of standard drinks (SDs) consumed. An SD is defined by the amount of pure alcohol contained in a drink, and it varies among countries [14,16,17]. For example, in Australia, an SD contains 10 g of pure alcohol, in the United Kingdom and Iceland, it contains only 8 g, whereas in Austria it is 20 g [17]. It is deemed risky drinking if alcohol consumption is more than 5 SDs for men and 4 for women on a single occasion [18]. If total weekly alcohol consumption is greater than or equal to 15 SDs for men and 13 for women in the United States or over 14 SDs for men and 9 for women in Sweden, it is also considered as risky drinking [19-21]. Risky drinking can also be measured by scales such as fast alcohol screening test (FAST), alcohol use disorders identification test (AUDIT), and AUDIT for consumption (AUDIT-C) by scoring 3 or higher in FAST [22], over 8 for men and 6 for women in AUDIT [23], or 4 for men and 3 for women in AUDIT-C [24].

The other major type of UAU is AUD. It is a chronically recurrent brain impairment in which compulsive and maladaptive alcohol use results in behavior dysregulation and negative mood once alcohol consumption is ceased [16,25]. Alcohol abuse and alcohol dependence are 2 major representatives for moderate and severe degrees of AUD, respectively [16,25,26]. Consumers with either of them can suffer from adverse consequences. Alcohol abuse, that is, unrestrained alcohol use, can make consumers fail to meet their major obligations and cause or exacerbate health and social problems [16,27]. More seriously, alcohol dependence, that is, a constant and strong desire for alcohol use without self-control or consideration of health, might result in physical or mental health problems once a large amount of alcohol is consumed over a long period [28]. To be diagnosed with AUD, a person should meet at least two of the 11 criteria listed in the Diagnostic and Statistical Manual of Mental Disorders 5th Edition in 1 year [29].

Mobile health (mHealth), also known as ecological momentary intervention [30,31], refers to the use of mobile devices, such as mobile phones, personal digital assistants, or other wireless devices, to deliver medical or public health services in a timely manner and in real-world living settings [30,32]. Due to limited human resources available for delivering continuous health care services for community-dwelling consumers suffering from chronic diseases, mHealth interventions are increasingly considered by the decision makers as a potential alternative solution in providing same quality but low-cost services [33]. Similarly, there has been a growing interest in using mobile phones to deliver public health interventions to support consumer self-control of UAU.

mHealth interventions are mainly delivered solely or in combination of 3 channels: short message services (SMS) text messaging, apps, and interactive voice response (IVR). SMS text messaging has been used to guide consumers to change alcohol use behavior, for example, to reduce alcohol intake to enable self-control of UAU [19,34]. Apps have been used to monitor consumers’ alcohol use and to provide visual feedback about drinking behavior based on statistical analysis of input data. Raising self-awareness can ignite consumers’ self-regulation so as to reduce alcohol use [35,36]. IVR has been used to generate audial interactions and to provide automatic answers to consumer queries on UAU [37,38]. Therefore, these 3 delivery channels can all provide effective and efficient interventions for consumer self-control of UAU.

### Objectives

Recent reviews on digital interventions for self-control of UAU focus on the benefit of such interventions on improving health care services. In total, 2 reviews investigated electronic or Web-based interventions and found that despite a small effect, these interventions might improve behavioral outcomes, particularly for the group less likely to access traditional alcohol interventions such as women, youth, and risky drinkers [39,40]. A total of 5 reviews narrowed down the scope on mHealth interventions for self-control of UAU. In total, 2 of them focused on SMS text messaging and found that although the behavioral outcomes were modest, it was still a worthwhile endeavor [41,42]. The other 3 reviews suggested mHealth interventions, especially the ones that can provide personalized feedback, were beneficial for the reduction of UAU with their high fidelity, anonymity, and accessibility [31,43,44]. However, as the mHealth interventions were still nascent in nature, there is still a lack of understanding about how such interventions really work for changing UAU. Solid evidence about the efficacy of these interventions from empirical field trials is required. Moreover, the other health outcomes, such as physiological and cognitive outcomes, need to be studied. Therefore, this review aimed to synthesize and understand the research evidence about the efficacy of mHealth interventions on various health outcomes for consumer self-control of UAU and to identify the core components to achieve these outcomes.

## Methods

### Study Design

A mixed-methods systematic review was conducted. Literature search and screening followed the preferred reporting items for systematic reviews and meta-analyses [45]. Data extraction was guided by the Consolidated Standards of Reporting Trials-electronic health checklist (V.1.6.1) [46]. The methodological quality of the studies was assessed by the Mixed Methods Appraisal Tool (MMAT) [47]. Data synthesis and analysis followed the principle of realist synthesis [48] and qualitative content analysis [49].

### Literature Search and Screening

The literature search was performed from December 2016 to March 2017 and further refined in August 2018 in 7 electronic interdisciplinary databases: Scopus, PubMed, PubMed Central, CINAHL Plus with full text, MEDLINE with full text, PsycINFO, and PsycARTICLES (see Multimedia Appendix 1). The following terms and medical subject headings were used individually or in combination to identify the relevant publications: “mHealth,” “text message,” “SMS,” “App,” “IVR,” “self-control,” “self-regulation,” “alcohol*,” and “intervention.” To ensure adequate coverage, a manual search was also conducted to identify papers from *Journal of Medical Internet Research* and its sister journals. The search was restricted to peer-reviewed journal papers published in English between 2008 and 2017. In addition, the following criteria were used in the selection of papers.

#### Inclusion Criteria

The papers were included in which (1) the research focused on supporting consumer self-control of UAU; (2) health intervention was delivered through mobile phone technologies; and (3) the data were collected from empirical randomized controlled trials.

#### Exclusion Criteria

The papers were excluded that (1) reported clinical therapy such as injection and medication rather than consumer active participation in the daily self-control of UAU; (2) did not report any alcohol-related health outcome; (3) used the intervention not dealing with UAU or containing Web-based components delivered by desktop or Web-based computer applications; or (4) were review papers, study protocols, conceptual papers, editorials, government reports, or guidelines in the topic area.

### Data Extraction

Data were extracted using a combination of an Endnote X8 and an Excel spreadsheet by 2 authors independently. These included name(s) of the author(s), year of publication, country of origin, population type, study setting, type of UAU, study type, eligibility, sample size, study arms and grouping, nonmobile cointervention, mHealth intervention theory, delivery mode, mHealth intervention content, implementation procedure, measurement, and outcomes.

### Quality Assessment of the Studies

All studies were assessed using the 4 criteria in section 2 of the MMAT, in terms of (1) randomization or sequence generation, checking if there is a clear description about randomization; (2) allocation concealment, verifying if there is a clear description about blinding; (3) outcome data, confirming if more than 80% outcomes were reported; and (4) attrition, assessing if less than 20% of the participants dropped out. Responses to each criterion were “yes,” “no,” or “can’t tell.”

### Data Synthesis and Analyses

Data were synthesized and analyzed using an inductive method. We reviewed all data that collected and identified similar notions and tagged them with the same code. Thereafter, we grouped the codes with similar meaning into an overarching concept. Concepts with similar meaning were grouped into a category that addresses our research question. The coding and data management were iteratively developed through constant comparison of the similarities and differences among codes.

To explore the initial outcomes about which components really make the intervention works, chi-square testing was conducted to test the relationship between health outcomes with the following 4 parameters: (1) types of UAU, being risky drinking or AUD; (2) with or without nonmobile cointervention; (3) theory-based or not; (4) deployment setting, being clinical, educational, or community based.

## Results

### Search Outcome

**Figure 1:**
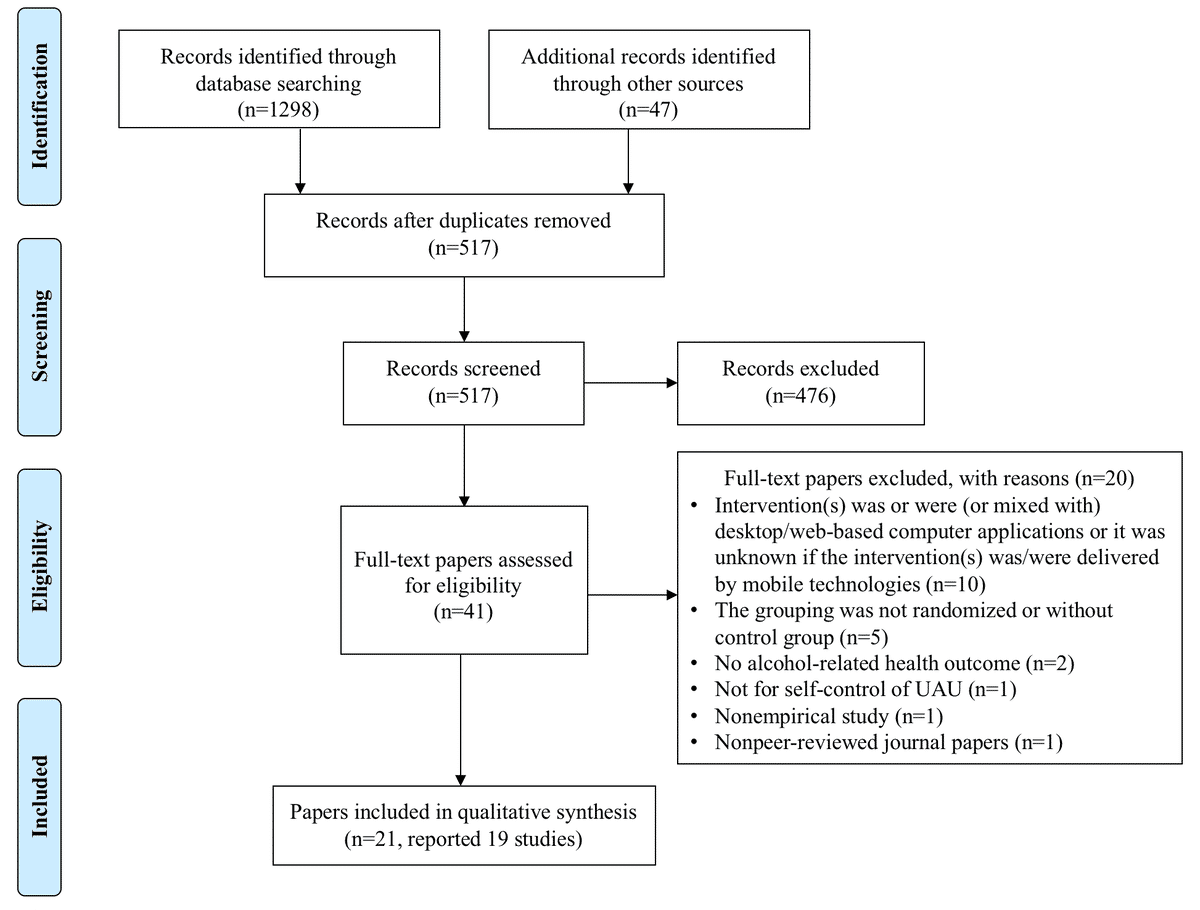
Literature search and screening process.

The primary search yielded 1345 publications. After removing duplicates, 517 papers remained. Their titles and/or abstracts were manually screened against the inclusion and exclusion criteria. This led to 41 candidate papers. Of these, 20 were excluded after further scrutinizing the full paper (see Multimedia Appendix 2). Finally, 21 papers were included. Of these, 4 papers were from 2 studies. Suffoletto et al published 2 papers based on the same study population in 2014 and 2015 [50,51], respectively; so did Agyapong et al in 2012 and 2013 [52,53]. Therefore, a total of 19 studies were eligible for review (see Figure 1 and Multimedia Appendix 3). Among these studies, 58% (11/19) met all 4 MMAT criteria [19,34,37,38,52-59] and 32% (6/19) met 3 criteria [20,21,35,50,51,60,61], indicating high methodological quality in 90% of these studies (see Multimedia Appendix 4).

### Characteristics of Studies

Although we searched studies published since 2008, all 19 eligible studies were conducted in 2012 and beyond and were from 7 developed countries (see Multimedia Appendix 5).

Of these, 9 studies (9/19, 47%) were conducted in the United States [19,34,35,38,50,51,55,57,59,62], 8 in Europe [20,21,37, 52-54,56,58,61], and only 2 in New Zealand [60,63].

Study arms ranged from 2 to 6. In total, 12 studies (12/19, 63%) were 2-arm trials with an intervention arm and a control arm [21,34,35,52,53,55,56,58-63]. The control-arm participants received (1) no intervention [35,56,58]; (2) nonmobile intervention with the same content through interview [55,62], email [21], and e-booklet [61]; (3) nonalcohol-related content [34,52,53] or only assessment for monitoring purpose [60,63] through the same mobile devices; or (4) different rewarding mechanisms for their abstinence [59]. A total of 5 studies (5/19, 26%) had 3 arms. Of these, 2 added an assessment-only arm besides the intervention and control arms [50,51,57]. Hasin et al employed an arm in which the participants only received intervention through interview [38]. Gajecki et al used 2 intervention arms delivered by 2 different mobile apps in 1 study [20] and 2 intervention arms that started to use the app at different times in another study [54]. In the last 2 studies (2/19, 11%), Andersson conducted a 5-arm trial in which an mHealth intervention was compared with Web-based intervention and nonintervention. Both the mHealth and Web-based interventions had 2 implementation procedures, single and repeated [37]. Muench et al employed a 6-arm design, including 1 nonintervention arm, 1 assessment-only arm, and 4 intervention arms containing different contents [19].

**Figure 2:**
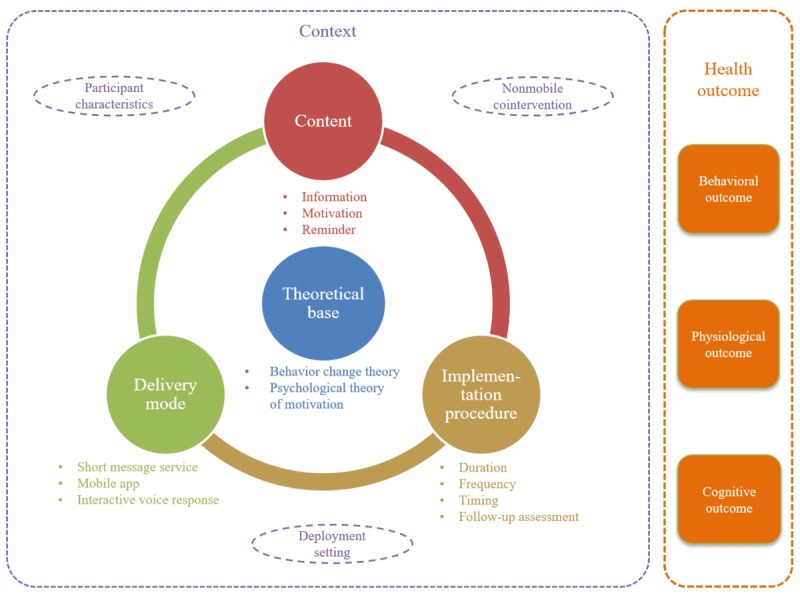
In total, 5 core components of mobile health interventions for self-control of unhealthy alcohol use and 3 types of potential health outcomes.

We identified 5 core components of mHealth interventions for UAU: context, theoretical base, content, delivery mode, and implementation procedure and 3 types of potential health outcomes: behavioral, physiological, and cognitive outcome (see Figure 2 and Multimedia Appendix 6).

### Five Core Components of Mobile Health Interventions for Self-Control of Unhealthy Alcohol Use

#### Context

There are 3 types of contexts: participant characteristics, deployment setting, and nonmobile cointervention, which were conducted simultaneously to support the mHealth intervention. 

The participants can be categorized into 2 cohorts according to their age: youth group aged below 35 years [20,21,34,37,50, 51,54,57,59,60,62,63] and middle and old aged group, aged above 35 years [19,35,38,52,53,55,56,58,61]. They were either risky drinkers [19-21,34,37,38,50,51,57,59-63] or had AUD [20,35,38,52-56,58]. They suffered from comorbidity of depression [52,53], HIV [38,55], drug dependence [55], or smoking [57]. The interventions were deployed in educational settings [20,21,34,37,54,57,60,62,63], clinical settings [35,38,50-53,56,58], and community-based settings [19,55,59, 61]. The nonmobile cointervention included social intervention guided by the theory of motivational interviewing [38,50,51,55,57,62] and paper-based intervention in which participants were provided with guidelines for safe alcohol use about the mHealth intervention [19].

#### Theoretical Base

In total, 2 types of theories were reported to guide the design and implementation of the mHealth interventions, including behavioral change theories and psychological theories of motivation.

Behavioral change theories included theory of planned behavior [20,21,50,51,56,63], health belief model [19,50,51], social cognitive theory [21,63], theory of reasoned action [50,51], information motivation behavioral model [50,51], cognitive behavioral therapy [57], and social learning theory [19]. Psychological theories of motivation included self-determination theory [21,35,63], model of action phases [21,63], and contingency management [59]. Notably, although Aharonovich et al did not report any explicit theory applied to their intervention, the design of their app, HealthCall, was theory based [55].

#### Delivery Mode

A total of 3 delivery modes were identified: SMS text messaging (12/19, 63%) [19,21,34,50-53,56,58-63], app (5/19, 26%) [20,35,54,55,57], and IVR (2/19, 11%) [37,38]. In total, 6 apps tested in the 5 studies were TeleCoach [54], Brief Alcohol and Smoking Intervention for College Students via Mobile (BASICS-Mobile) [57], HealthCall-S [55], Alcohol-Comprehensive Health Enhancement Support System (A-CHESS) [35], PartyPlanner, and Promillekoll [20]. Only 2 studies described the underlying operating system for these apps [20,57]. PartyPlanner and Promillekoll ran on the Android or iOS [20], and BASICS-Mobile ran on Blackberry, Android, or iOS [57].

#### Content

In total, 3 types of content were designed to support the participants’ self-control of UAU. They were information [19-21,34,35,37,38,50-58,60-63], motivation [19,21,34,35,37, 50-53,55-57,59,61,62], and reminder [19,35,50,51,55,56,58,59].

Informational content included general and personalized information. The general information facilitated the participants in (1) enriching their knowledge about risks and negative consequences of UAU [19,34,37,50,51,60,61,63], alcohol-related facts [34,37,54,57,61], social drinking norms [50,51,62], and benefits of reducing drinking amount according to safety guidelines [19]; (2) acquiring strategies to control alcohol use [20,34,50,51,54,55,57,58,62], to handle relapse or cravings [52-54,57,61], to manage emotion [54,61], and to reduce intoxication [37]; (3) getting referrals such as alcohol counseling services [55,58], instant library, and weblinks to further alcohol-related information [35]; and (4) conducting recommended actions for self-control of UAU such as tracking and reporting their drinking facts [19,20,35,37,38, 50,51,54-56,58,62,63], reasons for drinking or abstinence [38,55], and estimated blood alcohol concentration (eBAC) value [20], mood [38], medication adherence [38], and well-being [38]; introspecting their performance [21]; or simulating a drinking occasion to set personal goal of eBAC and comparing actual eBAC after drinking against this goal [20].

The personalized information helped the participants in (1) providing the tailored feedback according to their responses [19,37,50,51,55,57]; (2) recommending them to set intermittent low-risk drinking goals [61] to replace drinking alcohol by alternative activities [37,57], to celebrate goal attainment [50,51,61], to self-reflect on challenges of UAU [21], to improve the drinking plan, and to reinforce self-control behavior [37,50,51]; and (3) addressing their problems identified at various stages [19].

Motivational content included (1) encouragement messages for reducing alcohol use [21,34,37,50-53,57,61,62], committing to preset drinking goals [19,50,51,55,56] and medical adherence [52,53], and releasing distress [35]; (2) peer support through sharing experiences with others in the anonymous discussion groups [35]; and (3) possible monetary compensation to incent participants to submit their valid on-time video [59].

Reminding content facilitated participants in (1) reminding them to remember and fulfill their promises [50,51,55,56,58,59] and (2) warning them about alcohol risks at their risky drinking times [19] or when they were near high-alcohol places detected by global positioning system [35].

#### Implementation Procedure

The duration of the interventions varied, ranging from 4 days [62], 1 week [37,60,63], 2 weeks [57], 4 weeks [21,37,59], 6 weeks [34], 7 weeks [20], 2 months [38,55,58], 3 months [19,50-54], 6 months [56,61], to 8 months [35].

With regard to the frequency, SMS text messages were sent once [34], twice [50,51,58], or 4 times [21,60] weekly in 5 studies and once [63], twice [52,53], 1 to 3 times [59], or 4 to 6 times [62] daily in 4 studies. The frequency appeared to reduce when the length of the study increased [56,61]. Haug et al sent 1 SMS text message per week in the first 8 weeks and then 1 per fortnight in the remaining 18 weeks [56]. Brendryen et al sent 1 SMS text message per day for 8 weeks, then 1 per week for 4 weeks, and finally 1 per month in the last 2 months [61]~Brendryen, 2014 #8^. Muench et al sent the SMS text messages with different content at a different frequency, either once daily for educating participants about alcohol use or once weekly for self-monitoring content and feedback [19]. The participants in Alessi and Petry’s study were given a breathalyzer and the corresponding accessories to self-measure breath alcohol concentration (BrAC) and submit a valid real-time video containing the whole self-measuring process to the organizer via SMS text message 1 to 3 times per day at the fixed time interval to prove their abstinence [50]. The intervention-arm participants would be rewarded with more vouchers if their BrAC value was normal. In contrast, the control-arm participants were not rewarded although their BrAC value was normal [50].

In 3 studies in which the interventions were delivered by mobile apps, the frequency of data collection was once daily in Aharonovich et al’s study [55] and once weekly in Gustafson et al and Gajecki et al’s study [35,54]. In most cases, apps were used in real time according to a participant’s preference, typically to receive a certain recommendation once a preset condition was met. For example, Promillekoll could send real-time notification and the corresponding strategies to control alcohol use if a participant’s eBAC was over 0.06% [20]. A-CHESS would send an alarm when a participant was near a high-risk alcohol place to be detected by the embedded global positioning system [35].

Andersson divided his intervention arm into 2 subgroups, both receiving the same content but through different delivery modes, either delivering single IVR every day for 1 week or delivering repeated IVR for 4 weeks [37]. Hasin et al requested their intervention-arm participants to spend 1 to 3 min per day to send back their answers to a series of questions, asking their compliance with drinking guidelines on the previous day via a toll-free number [38]. The participants’ phone calls were initially answered by the prerecorded IVR in the first 30 days. After evaluating a participant’s IVR data, the consultant reset the person’s drinking goal for the next 30 days [38].

In terms of timing of delivery, 10 studies reported the fixed time or time slots to deliver the intervention, the rest were flexibly available on demand. The popular days of intervention delivery were Thursday [34,50,51,58,60,63], Saturday [21,34,59,60,63], and Sunday [21,50,51,59,63], mainly at or after 6 pm [19,34,52,53,56,59,60,63].

All studies conducted the baseline assessments. In total, 6 studies conducted an assessment during the intervention period to explore the initial outcome [35,38,54,55,58,61]. The postintervention assessments were conducted in all studies at different time points with different numbers of repetitive measurement. A total of 17 studies conducted 1 assessment immediately after the intervention [19-21,34,35,38,50-61,63]. A total of 8 studies conducted the second assessment 1 month [38,57], 6 weeks [34], 1 academic semester [60,63], 3 months [50-53], and 4 months [35] after the intervention. In total, 2 studies conducted the third assessment 3 [50,51] and 4 months [38] after the intervention. Only 1 study conducted the fourth assessment after 10 months of the intervention [38]. Instead of immediately measuring the outcomes, in 2 studies, the measures were conducted only after 1 month [62]. Of these, 1 study measured the outcome 4 weeks after the intervention for the single IVR intervention arm and 1 week after the intervention for the repeated IVR intervention arm [37].

### Health Outcomes

#### Behavioral Outcome

Behavioral outcome was measured in 18 studies [19,20,34,35,37,38,50-63]. Significant positive outcome was found in 11 of these studies [19,35,37,38,50-54,59-61,63]. These positive outcomes were measured by 1 or more indicators. These included the decreased number of SDs [19,50,51,54,61,63], heavy drinking days [19,35,38,50,51,59], RSOD or binge drinking prevalence [50,51,54], alcohol-related injury prevalence [50,51], and peak eBAC value [37]; increased number of abstinence days [19,52,53,59] or the increased negative affect score in Alcohol Abstinence Self Efficacy Scale [52,53]; and the decreased score in the Alcohol Addiction Severity Index, Drinker Inventory of Consequences [59], or AUDIT [37].

No significant behavioral change was found in 6 studies [34,55-58,62]. In total, 2 studies reported a gender-related behavioral outcome [20,63]. Contrary to the initial objective of reducing UAU, the male participants in the intervention arm significantly increased drinking frequency, whereas no change was found in the female participants and the control arm in 1 study [20]. In the study conducted by Riordan et al, after providing intervention-arm participants with 1-week SMS text messages, the female participants consumed significantly less alcohol 1 week and 1 semester later than their female counterparts in the control arm. However, no intervention effect was found for the male participants [63].

#### Physiological Outcome

Physiological outcome was measured in only 1 study via BrAC [59]. Alessi and Petry found a significant improvement in the percentage of negative BrAC in the intervention group but no significant change in the control group [59].

#### Cognitive Outcome

Cognitive outcome was measured in 3 studies [21,34,62] and was significantly positive in only 1 study in which the participants’ readiness to change UAU behavior in the intervention arm was significantly improved [62]. No significant cognitive change was found in the other 2 studies in terms of motivation to change and self-confidence to resist alcohol [21,34].

### Comparison With the Differences in Health Outcomes Among Different Groups of Studies

Over half of the SMS- and IVR-enabled interventions were effective in reducing alcohol use or increasing readiness to change UAU in 8 out of 12 studies (67%) [19,50-53,59-63] and 2 out of 2 studies (100%) [37,38], respectively. In contrast, app-enabled interventions were only successful in reducing alcohol use in 2 out of 5 studies (40%) [35,54].

Chi-square test did not find any significant differences in health outcomes among groups of studies with different conditions. It suggested that the health outcomes were similar regardless of the types of UAU studied, whether there was nonmobile cointervention, whether the study was theoretical-based, or which setting it was deployed.

## Discussion

### Principal Findings and Comparison With Previous Work

This study aimed to synthesize and understand the research evidence about efficacy of mHealth interventions on different health outcomes for consumer self-control of UAU and to identify their core components to achieve these outcomes. In total, 19 studies were systematically reviewed and 3 types of health outcomes such as behavioral, physiological, and cognitive outcome and 5 components of these interventions such as context, theoretical base, delivery mode, contents, and implementation procedure were found.

#### Health Outcomes

As approximately two-thirds (11/18) of the studies that measured the behavioral outcomes identified a significant positive change [19,35,37,38,50-54,59-61,63]: mHealth interventions appear to be more effective in changing UAU behavior in comparison with the traditional methods. The results could be explained by the information-motivation-behavioral skills model, which suggests that a participant’s behavior change is attributed to the provided information, motivation, and improved skills [64]. This is also in accordance with the findings of Regmi et al’s review in smoking cessation context where the abstinence days of smoking increase after applying mHealth interventions [65].

Despite the significant 100% positive physiological outcome measured by BrAC, only 5% of the included studies assessed the physiological measurement [59]. There might be two reasons for this. First, people tend to test their biomedical markers in hospital or clinic rather than by themselves as they might lack corresponding skills and it is inconvenient. Second, the corresponding self-testing devices are not cheap and not all research projects can afford them, especially for the projects with a large sample size. Instead, researchers preferred to measure behavioral outcomes because the rough BAC can be calculated simply using Widmark formula once a participant reports his or her alcohol use [66]. In addition, the unique factor to affect the BrAC is alcohol intake. Therefore, the physiological outcome must change when the behavior changes.

For the same reason, as the cognitive outcomes are inconvenient to measure in comparison with behavioral ones, only 16% of the studies [21,34,62] assessed cognitive changes, of which 33% [62] were significantly improved. This might be because cognition can be influenced by various factors, and their measurement can be somewhat subjective and abstract. For example, Mason et al assessed cognitive change using 5 variables: alcohol expectations, readiness to change drinking behavior, importance of change, confidence in ability to change, and intentions to reduce alcohol use. Only the variable of readiness to change drinking behavior was improved [62]. Notably, although all these 3 studies also reported the improvement in behavior changes, it is still not enough to conclude that behavior always changes with cognition. According to the theory of cognitive dissonance proposed by Leon Festinger, a person can be motivated to reduce own psychological inconsistency and discomfort by changing the behavior [67]. No matter whether the interventions were genuinely accepted by the participants or not, most of them modified their behavior in compliance with the information they received from the interventions to reduce their interconflicts [67]. Therefore, when their cognitive changes were assessed, they might provide the real thoughts, which might not be consistent with the behavior that were displayed.

Complementing the traditional interventions such as face-to-face counseling, in which unhealthy alcohol users’ access to treatment was provided in a passive manner within a confined time and location, mHealth interventions open new opportunities for engaging consumers in positive self-control with increased flexibility. The effect of control was improved by continuous tracking and monitoring, interactive communication, or personalized feedback from health care providers anytime, anywhere [30,68,69].

#### Five Components of Mobile Health Interventions for Self-Control of Unhealthy Alcohol Use

Participants in most reviewed studies were risky drinkers without documented pathological conditions [19-21,34,37,38, 50,51,57,59-63]. We did not find much difference in the intervention outcome between the types of participants, being risky drinker or AUD. This result is consistent with the finding of Blow et al that health outcomes of an intervention are not influenced by the level of severity of alcohol addiction [70]. However, this is contradictory with the findings in the previous review conducted by White et al that e-interventions can be particularly useful for at-risk users [40]. Kazemi et al also seconded that for this population group, mHealth intervention might be the most cost-effective UAU management strategy [71]. The paradox might be caused by the different conditions such as timing and frequency of the interventions or different population types and settings [42].

The gender difference in intervention outcome found in 2 studies [20,63] might be explained by the observation of Hirschi and Gottfredson that men have lower self-control than women [72]. Notably, these 2 studies were both done on young adult students in university settings. This might suggest that it is much more difficult for males in this setting to change their behavior in terms of UAU. First, there are strong social or peer norms in this cohort, which prevent the change of drinking behavior [73], and second, males seem to be less compliant and agreeable than females, and they lack ability to absorb the meaning of the SMS text messages [74,75]. Riordan et al offered some suggestions for improving SMS text messaging for young men and later demonstrated that using more colloquial tone and sending only messages with the potential social consequences of UAU are better for this population [60]. Similar to the finding of Platt et al [76], we did not find any significant relationship between the health outcome and deployment setting.

Although not having any significant impact on health outcomes, cointervention, such as induction or training to enable a participant to confidently use the apps or IVR, is an integral, vital component for a successful mHealth intervention [77,78]. This might explain why more cases of nonmobile cointervention were reported in interventions delivered via apps (3/5, 60%) and IVR (1/2, 50%). Most likely, the participants were more familiar with SMS text messaging than the other 2 delivery modes; therefore, the cointervention was less reported in the studies delivered by SMS text messaging (3/12, 25%). Notably, the population of the 2 studies in which interventions were delivered via apps without formal reporting of cointervention was university students at a younger age. This might be because of the internet use and mobile phone technologies are popular in this cohort; thus, the app designers did not consider it necessary to provide the students with training to use the app [79].

Behavior change theory provides the foundation for the formation of strategies to incrementally change a consumer’s behavior of UAU [80]. Psychological theory of motivation is used to develop motivational strategies to control UAU against psychological craving for alcohol [81]. Although mHealth interventions based on theory can improve instructional design and the effect of self-control of UAU [76], no significant difference in health outcomes was found in this review for the studies based on theory and those otherwise, which is in accordance with the finding of Garnett et al [82]. There might be two reasons to explain this phenomenon. First, from what was described in the Methods, it appears that theory was implicitly applied to the mHealth interventions although a study might not make the claim to be theory based. For example, Bock et al did not report the use of any theory; however, one of the SMS text messages in their intervention “always have an exit plan” indicated the unconscious application of the theory of planned behavior [34]. Second, it takes time to bring in tangible health outcomes for participant’s self-control of UAU [52,53].

Almost all SMS- or IVR-enabled interventions were effective in reducing alcohol use or increasing readiness to change except the mobile apps [20,55,57]. This might be because the former 2 types of interventions were delivered proactively, on regular basis, always accessible to the participants regardless of their intention. In contrast, the participants’ access to the app-based interventions relied on their self-action of opening the apps, which might not always happen. This is consistent with the findings in Meredith et al’s review [83], and it also recommends that the future mHealth apps need push notifications regularly to prompt the active engagement of the users.

Informational content facilitated the participants to develop essential knowledge and skill to build their capacity to change their belief and UAU behavior. It also provided necessary feedback to enable self-awareness of UAU status, which could help execute self-regulation of UAU. Motivational content provided continuous encouragement and peer support through experience sharing to raise the participants’ morale in changing UAU behavior. Reminding content provided constant recall to ensure the participants to stay on track in self-control of UAU. Delivery of these 3 types of content is in line with the model of human practical reasoning developed by Michael Bratman [84].

As the length of the reviewed studies was not long enough, ranging from 4 days to 8 months, it is no surprise that there was no obvious improvement in tangible health outcomes in many studies. Longer duration, that is, 6 months or more [35,61], more frequent delivery [52,53,59,62] and certain techniques such as tangible incentives [62], and assessment during the intervention [61] might help achieve positive outcomes. In contrast, a relatively small sample size, less than 100 [34,55,56] and a short follow-up period, less than 2 months [57], might cause a lack of significant health outcomes for the interventions. However, whether the health outcomes can be influenced by these factors still needs to be verified.

With the same content and implementation procedure, Andersson et al found differences in health outcomes measured by peak eBAC and AUDIT scores with different delivery modes in which the efficacy was better delivered by IVR than the Web [37]. Similarly, with the same delivery mode and implementation procedure but different content, Muench et al also found differences in health outcomes measured by numbers of SDs, heavy drinking days, and abstinent days. The content that highlighted the negative consequences of UAU was significantly more likely to bring about positive health outcomes than the content that emphasized the benefits of UAU abstinence [19]. Furthermore, with the same content and delivery mode, Gajeck et al found that the health outcomes measured by SD and drinking frequency were significantly different with different intervals of intervention [54].

Although the first generation of iPhone was released in June 2007, marking the debut of smartphone technology [85], no eligible studies were found before 2012. It appears that using mobile phones to deliver mHealth interventions for UAU was staged in 2012.

### Limitations

The first limitation of this study was that the coverage of the studies might not be exhaustive, because of which our search was confined to the 7 databases. However, the comprehensiveness of these databases can ensure the representativeness of the trend suggested by this study. The heterogeneity of participant characteristics, intervention, and health outcome measures makes it difficult to compare rigorously the findings among the studies. A lack of homogenous, quantitative measures in the original studies also deemed it impossible to conduct more rigorous meta-analysis. As only peer-refereed journal papers were included to ensure the rigor of this study, there could be a potential risk of reporting bias toward positive findings.

### Conclusions

This systematic review summarized the extant research evidence about the health outcomes of mHealth interventions for consumer self-control of UAU. A total of 3 health outcomes, that is, physiological, behavioral, and cognitive outcomes and 5 core components of these interventions, that is, context, theoretical base, delivery mode, content, and implementation procedure, were synthesized and analyzed. In comparison with the traditional interventions, the evidence to support effectiveness of mHealth interventions for consumer self-control of UAU is modest at best. A majority of studies showed that mHealth interventions brought positive health outcomes in helping unhealthy alcohol users to proactively engage in self-control of their UAU behavior, especially for the ones delivered by SMS text messaging and IVR systems. Sound evidence is yet to be sought about the effects of these interventions in improving the physiological and cognitive outcomes. Further research is needed to gather evidence about the optimal design of mHealth interventions, their implementation, and effects in supporting consumer self-control of UAU.

## Appendix

Multimedia Appendix 1 Search and screen records.

Multimedia Appendix 2 Excluded papers with reasons.

Multimedia Appendix 3 List of included studies.

Multimedia Appendix 4 Quality appraisal of the included studies.

Multimedia Appendix 5 Characteristics of the included studies.

Multimedia Appendix 6 Five components and three types of health outcomes.

## Abbreviations

A-CHESS: Alcohol-Comprehensive Health Enhancement Support System
AUD: alcohol use disorder
AUDIT: alcohol use disorders identification test
AUDIT-C: alcohol use disorders identification test for consumption
BASICS-Mobile: Brief Alcohol and Smoking Intervention for College Students via Mobile
BrAC: breath alcohol concentration
eBAC: estimated blood alcohol concentration
FAST: fast alcohol screening test
IVR: interactive voice response
mHealth: mobile health
MMAT: Mixed Methods Appraisal Tool
RSOD: risky single-occasion drinking
SD: standard drink
SMS: short message services
UAU: unhealthy alcohol use
