# Supplementary material for: Mobile Health Interventions for Self-Control of Unhealthy Alcohol Use: Systematic Review
Source: JMIR Mhealth Uhealth. 2019 Jan 29;7(1):e10899. doi: 10.2196/10899 (PMC6371076; doi:10.2196/10899)
Supplement: Multimedia Appendix 1 [file mhealth_v7i1e10899_app1.pdf]

Appendix 1. Searching and screening records

Database

Scopus, MEDLINE with Full Text, CINAHL Plus with Full Text, PsycINFO, PsycARTICLES, PubMed, PMC

Track

Step 1

Scopus

	Searching terms and combinations	Searching field	Limit
	alcohol OR alcohol*	Title, abstract, keywords	Date range (inclusive): 2008 to 2017 Document type: article Language: English
AND	mHealth OR m-Health OR "mobile health" OR "text messag*" OR "short message servic*" OR SMS OR "mobile phone" OR ((mobile OR smartphone OR cellphone) AND (app OR app* OR tech*)) OR "interactive voice response" OR IVR	Title, abstract, keywords	
AND	Interventio* OR (treatment AND program*) OR self-* OR "self control" OR "self regulation"	Title, abstract, keywords	
**Result**			**360**

MEDLINE with Full Text, CINAHL Plus with Full Text, PsycINFO & PsycARTICLES

	Searching terms and combinations	Searching field	Limit
	alcohol OR alcohol*	Abstract	Date of publication: January 2008 to December 2017 Source type: academic journals Language: English
AND	mHealth OR m-Health OR "mobile health" OR "text messag*" OR "short message servic*" OR SMS OR "mobile phone" OR ((mobile OR smartphone OR cellphone) AND (app OR app* OR tech*)) OR "interactive voice response" OR IVR	Abstract	
AND	Interventio* OR (treatment AND program*) OR self-* OR "self control" OR "self regulation"	Abstract	
**Result**			**628 (Medline 286, PsycINFO 182, CINAHL 146, PsycARTICLES 14)**

Pubmed

Search code	Query	Results
#1	Search **(alcohol[Title/Abstract] OR alcohol*[Title/Abstract])**	304392
#2	Search **(mHealth[Title/Abstract] OR m-Health[Title/Abstract] OR "mobile health"[Title/Abstract] OR "text messag*"[Title/Abstract] OR "short message servic*"[Title/Abstract] OR SMS [Title/Abstract] OR "mobile phone"[Title/Abstract] OR ((mobile[Title/Abstract] OR smartphone[Title/Abstract] OR cellphone)[Title/Abstract] AND (app[Title/Abstract] OR apps[Title/Abstract] OR applicatio*[Title/Abstract] OR technology[Title/Abstract] OR technologies))[Title/Abstract] OR "interactive voice response" [Title/Abstract] OR IVR[Title/Abstract])**	1669
#3	Search **(Intervention[Title/Abstract] OR interventions[Title/Abstract] OR (treatment[Title/Abstract] AND program*)[Title/Abstract] OR self-control[Title/Abstract] OR self-regulation[Title/Abstract] OR self-manag*[Title/Abstract] OR self-efficacy[Title/Abstract] OR self-help[Title/Abstract] OR self-monitor[Title/Abstract] OR self-report[Title/Abstract] OR "self control" [Title/Abstract] OR "self regulation"[Title/Abstract])**	97883
#4	Search **(("journal article"[Publication Type]) AND ("2008/01/01"[Date - Publication] : "2017/12/31"[Date - Publication])) AND "english"[Language]**	8487865
1 and 2 and 3 and 4	Search **((((("journal article"[Publication Type]) AND ("2008/01/01"[Date - Publication] : "2017/12/31"[Date - Publication])) AND "english"[Language])) AND ((Intervention[Title/Abstract] OR interventions[Title/Abstract] OR (treatment[Title/Abstract] AND program*)[Title/Abstract] OR self-control[Title/Abstract] OR self-regulation[Title/Abstract] OR self-manag*[Title/Abstract] OR self-efficacy[Title/Abstract] OR self-help[Title/Abstract] OR self-monitor[Title/Abstract] OR self-report[Title/Abstract] OR "self control" [Title/Abstract] OR "self regulation"[Title/Abstract]))) AND ((mHealth[Title/Abstract] OR m-Health[Title/Abstract] OR "mobile health"[Title/Abstract] OR "text messag*"[Title/Abstract] OR "short message servic*"[Title/Abstract] OR SMS [Title/Abstract] OR "mobile phone"[Title/Abstract] OR ((mobile[Title/Abstract] OR smartphone[Title/Abstract] OR cellphone)[Title/Abstract] AND (app[Title/Abstract] OR apps[Title/Abstract] OR applicatio*[Title/Abstract] OR technology[Title/Abstract] OR technologies))[Title/Abstract] OR "interactive voice response" [Title/Abstract] OR IVR[Title/Abstract]))) AND ((alcohol[Title/Abstract] OR alcohol*[Title/Abstract]))**	**278**

PMC

Search code	Query	Results
#1	Search **alcohol[Abstract]**	44193
#2	Search **(mHealth[Abstract] OR m-Health[Abstract] OR "mobile health"[Abstract] OR "text message"[Abstract] OR "text messaging"[Abstract] OR "short message servic*"[Abstract] OR SMS [Abstract] OR "mobile phone"[Abstract] OR ((mobile[Abstract] OR smartphone[Abstract] OR cellphone)[Abstract] AND (app[Abstract] OR apps[Abstract] OR applicatio*[Abstract] OR technology[Abstract] OR technologies))[Abstract] OR "interactive voice response" [Abstract] OR IVR[Abstract])**	6412
#3	Search **(Intervention[Abstract] OR interventions[Abstract] OR (treatment[Abstract] AND (program[Abstract] OR programme))[Abstract] OR self-control[Abstract] OR self-regulation[Abstract] OR self-manag*[Abstract] OR self-efficacy[Abstract] OR self-help[Abstract] OR self-monitor[Abstract] OR self-report[Abstract] OR "self control" [Abstract] OR "self regulation"[Abstract])**	53881
#4	Search **("2008/01/01"[Publication Date] : "2017/12/31"[Publication Date])**	2899881
1 and 2 and 3 and 4	Search **(((("2008/01/01"[Publication Date] : "2017/12/31"[Publication Date])) AND alcohol[Abstract]) AND ((mHealth[Abstract] OR m-Health[Abstract] OR "mobile health"[Abstract] OR "text message"[Abstract] OR "text messaging"[Abstract] OR "short message servic*"[Abstract] OR SMS [Abstract] OR "mobile phone"[Abstract] OR ((mobile[Abstract] OR smartphone[Abstract] OR cellphone)[Abstract] AND (app[Abstract] OR apps[Abstract] OR applicatio*[Abstract] OR technology[Abstract] OR technologies))[Abstract] OR "interactive voice response" [Abstract] OR IVR[Abstract]))) AND ((Intervention[Abstract] OR interventions[Abstract] OR (treatment[Abstract] AND (program[Abstract] OR programme))[Abstract] OR self-control[Abstract] OR self-regulation[Abstract] OR self-manag*[Abstract] OR self-efficacy[Abstract] OR self-help[Abstract] OR self-monitor[Abstract] OR self-report[Abstract] OR "self control" [Abstract] OR "self regulation"[Abstract]))**	**32**

Post-hoc searching from Journal of Medical Internet Research and its sister journals

	alcohol OR alcohol*	Abstract
AND	mobile OR SMS OR App OR IVR	Abstract
AND	Intervention OR self OR self-*	Abstract
AND	From 2008.01.01 to 2017.12.31	Publish date
Results		47

Total publications retrieved: 360+628+278+32+47=1345

Step 2.0

Excluding duplicates = 828

Record number of papers = 517

Step 3.0

Review titles and abstracts

Exclude papers = 476

Record number of papers = 41

Step 4.0

Review whole papers

Exclude papers = 20

Record number of papers = 21

Record number of studies = 19
