# Supplementary material for: Mobile Health Interventions for Self-Control of Unhealthy Alcohol Use: Systematic Review
Source: JMIR Mhealth Uhealth. 2019 Jan 29;7(1):e10899. doi: 10.2196/10899 (PMC6371076; doi:10.2196/10899)
Supplement: Multimedia Appendix 2 [file mhealth_v7i1e10899_app2.pdf]

Appendix 2. Excluded papers with reasons

Author (Year)	Title	Reason for exclusion	Defined criteria
You et al (2017)	Smartphone-based support system (SoberDiary) coupled with a Bluetooth breathalyser for treatment-seeking alcohol-dependent patients	Not randomized controlled trials	Inclusion criteria<br>(1) the research focused on supporting consumer self-control of UAU;<br>(2) the intervention was delivered through mobile technologies; and<br>(3) the data were collected from empirical randomized controlled trials.<br><br>Exclusion criteria<br>(1) reported clinical therapy such as injection and medication rather than consumer active participation in the daily self-control of UAU;<br>(2) did not report any alcohol-related outcome;<br>(3) used the intervention not dealing with UAU or containing web-based components delivered by desktop/web-based computer applications; or<br>(4) were review articles, study protocols, conceptual papers, editorials, government reports or guidelines in the topic area.
Tahaney & Palfai (2017)	Text messaging as an adjunct to a web-based intervention for college student alcohol use: A preliminary study	Contain web-based components	
Pedersen et al (2017)	A randomized controlled trial of a web-based, personalized normative feedback alcohol intervention for young-adult veterans	Contain web-based components	
Paz Castro et al (2017)	Moderators of outcome in a technology-based intervention to prevent and reduce problem drinking among adolescents	Contain web-based components	
Haug et al (2017)	Efficacy of a Web- and Text Messaging-Based Intervention to Reduce Problem Drinking in Adolescents: Results of a Cluster-Randomized Controlled Trial	Contain web-based components	
Hamilton et al (2017)	DIAMOND (DIgital Alcohol Management ON Demand): A mixed methods feasibility RCT and embedded process evaluation of a digital health intervention to reduce hazardous and harmful alcohol use	Feasibility study, did not report any alcohol-related outcome	
Glass et al (2017)	Treatment seeking as a mechanism of change in a randomized controlled trial of a mobile health intervention to support recovery from alcohol use disorders	Secondary analysis rather than empirical study	
Davies et al (2017)	Personalized digital interventions showed no impact on risky drinking in young adults: A pilot randomized controlled trial	Contain web-based components	
Bertholet et al (2017)	Smartphone application for unhealthy alcohol use: A pilot study	No control group	

Andersson et al (2017)	Interactive Voice Response with Feedback Intervention in Outpatient Treatment of Substance Use Problems in Adolescents and Young Adults: A Randomized Controlled Trial	Not self-control of UAU	
Sundström et al (2016)	Guided and unguided internet-based treatment for problematic alcohol use - A randomized controlled pilot trial	Contain web-based components	
Carrà et al (2016)	Impact of a Mobile E-Health Intervention on Binge Drinking in Young People: The Digital-Alcohol Risk Alertness Notifying Network for Adolescents and Young Adults Project	No control group	
Andersson (2015)	Smartphone applications can help in treatment for alcoholism	Not journal paper, but a commentary	
Gonzalez & Dulin (2015)	Comparison of a smartphone app for alcohol use disorders with an internet-based intervention plus bibliotherapy: A pilot study	Contain web-based components	
Hasin et al (2014)	HealthCall for the smartphone: technology enhancement of brief intervention in HIV alcohol dependent patients	No randomized	
Gamito et al (2014)	Executive functioning in alcoholics following an mhealth cognitive stimulation program: Randomized controlled trial	did not report any alcohol-related outcome	
Litt et al (2009)	Individualized assessment and treatment program for alcohol dependence: Results of an initial study to train coping skills	No control group	
Helzer et al (2008)	Using interactive voice response to enhance brief alcohol intervention in primary care settings	Not mobile	
Rose et al (2017)	A Randomized Controlled Trial of Brief Intervention by Interactive Voice Response	Not mobile	
Rose et al (2015)	Efficacy of automated telephone continuing care following outpatient therapy for alcohol dependence	Not mobile	
