# Supplementary material for: Mobile Health Interventions for Self-Control of Unhealthy Alcohol Use: Systematic Review
Source: JMIR Mhealth Uhealth. 2019 Jan 29;7(1):e10899. doi: 10.2196/10899 (PMC6371076; doi:10.2196/10899)
Supplement: Multimedia Appendix 3 [file mhealth_v7i1e10899_app3.pdf]

Appendix 3. List of included studies

1. Agyapong VI, Ahern S, McLoughlin DM, Farren CK. Supportive text messaging for depression and comorbid alcohol use disorder: Single-blind randomised trial. J Affect Disord 2012 Dec 10;141(2):168-176.PMID:22464008

   Agyapong VI, McLoughlin DM, Farren CK. Six-months outcomes of a randomised trial of supportive text messaging for depression and comorbid alcohol use disorder. J Affect Disord 2013 Oct;151(1):100-104.PMID:23800443

2. Aharonovich E, Stohl M, Cannizzaro D, Hasin D. Healthcall delivered via smartphone to reduce co-occurring drug and alcohol use in HIV-infected adults: A randomized pilot trial. J Subst Abuse Treat 2017 83:15-26.PMID:29129192

3. Alessi SM, Petry NM. A randomized study of cellphone technology to reinforce alcohol abstinence in the natural environment. Addiction 2013 May;108(5):900-909.PMID:23279560

4. Andersson C. Comparison of web and interactive voice response (IVR) methods for delivering brief alcohol interventions to hazardous-drinking university students: a randomized controlled trial. Eur Addict Res 2015 21(5):240-252.PMID:25967070

5. Bendtsen M, Bendtsen P. Feasibility and user perception of a fully automated push-based multiple-session alcohol intervention for university students: randomized controlled trial. JMIR Mhealth Uhealth 2014 2(2):e30.PMID:25098296

6. Bock BC, Barnett NP, Thind H, Rosen R, Walaska K, Traficante R, Foster R, Deutsch C, Fava JL, Scott-Sheldon LA. A text message intervention for alcohol risk reduction among community college students: TMAP. Addict Behav 2016 Dec;63(107-113.PMID:27450909

7. Brendryen H, Lund IO, Johansen AB, Riksheim M, Nesvåg S, Duckert F. Balance - a pragmatic randomized controlled trial of an online intensive self-help alcohol intervention. Addiction 2014 Feb;109(2):218-226.PMID:24134709

8. Gajecki M, Andersson C, Rosendahl I, Sinadinovic K, Fredriksson M, Berman AH. Skills training via smartphone app for university students with excessive alcohol consumption: A randomized controlled trial. International Journal of Behavioral Medicine 2017 24(5):778-788.PMID:28224445

9. Gajecki M, Berman AH, Sinadinovic K, Rosendahl I, Andersson C. Mobile phone brief intervention applications for risky alcohol use among university students: a randomized controlled study. Addict Sci Clin Pract 2014 9(1):11.PMID:24985342

10. Gustafson DH, McTavish FM, Chih M-Y, Atwood AK, Johnson RA, Boyle MG, Levy MS, Driscoll H, Chisholm SM, Dillenburg L. A smartphone application to support recovery from alcoholism: a randomized clinical trial. JAMA Psychiatry 2014 May;71(5):566-572.PMID:24671165

11. Hasin DS, Aharonovich E, O'leary A, Greenstein E, Pavlicova M, Arunajadai S, Waxman R, Wainberg M, Helzer J, Johnston B. Reducing heavy drinking in HIV primary care: a

randomized trial of brief intervention, with and without technological enhancement. Addiction 2013 Jul;108(7):1230-1240.PMID:23432593

12. Haug S, Lucht MJ, John U, Meyer C, Schaub MP. A pilot study on the feasibility and acceptability of a text message-based aftercare treatment programme among alcohol outpatients. Alcohol Alcohol 2015 Mar;50(2):188-194.PMID:25600249

13. Lucht MJ, Hoffman L, Haug S, Meyer C, Pussehl D, Quellmalz A, Klauer T, Grabe HJ, Freyberger HJ, John U, Schomerus G. A surveillance tool using mobile phone short message service to reduce alcohol consumption among alcohol-dependent patients. Alcohol Clin Exp Res 2014 38(6):1728-1736.PMID:24730528

14. Mason M, Benotsch EG, Way T, Kim H, Snipes D. Text messaging to increase readiness to change alcohol use in college students. J Prim Prev 2014 Feb;35(1):47-52.PMID:24114551

15. Muench F, van Stolk-Cooke K, Kuerbis A, Stadler G, Baumel A, Shao S, McKay JR, Morgenstern J. A randomized controlled pilot trial of different mobile messaging interventions for problem drinking compared to weekly drink tracking. PLoS ONE 2017 12(2):e0167900.PMID:28146560

16. Riordan BC, Conner TS, Flett JA, Scarf D. A brief orientation week ecological momentary intervention to reduce university student alcohol consumption. J Stud Alcohol Drugs 2015 Jul;76(4):525-529.PMID:26098027

17. Riordan BC, Conner TS, Flett JA, Scarf D. A text message intervention to reduce first year university students' alcohol use: A pilot experimental study. Digit Health 2017 Apr 26(3)PMID:29942597

18. Suffoletto B, Kristan J, Callaway C, Kim KH, Chung T, Monti PM, Clark DB. A text message alcohol intervention for young adult emergency department patients: a randomized clinical trial. Ann Emerg Med 2014 Dec;64(6):664-672.e664.PMID:25017822

    Suffoletto B, Kristan J, Chung T, Jeong K, Fabio A, Monti P, Clark DB. An interactive text message intervention to reduce binge drinking in young adults: a randomized controlled trial with 9-month outcomes. PLoS ONE 2015 Nov 18;10(11):e0142877.PMID:26580802

19. Witkiewitz K, Desai SA, Bowen S, Leigh BC, Kirouac M, Larimer ME. Development and evaluation of a mobile intervention for heavy drinking and smoking among college students. Psychol Addict Behav 2014 Sep;28(3):639-650.PMID:25000269
