# Supplementary material for: Mobile Health Interventions for Self-Control of Unhealthy Alcohol Use: Systematic Review
Source: JMIR Mhealth Uhealth. 2019 Jan 29;7(1):e10899. doi: 10.2196/10899 (PMC6371076; doi:10.2196/10899)
Supplement: Multimedia Appendix 4 [file mhealth_v7i1e10899_app4.pdf]

Appendix 4. Quality appraisal of the included studies

Author(s) (Year) [Reference]	Randomization or sequence generation	Allocation concealment	Outcome data	Attrition
Aharonovich et al. (2017)	Yes	Yes	Yes	Yes
Gajecki et al. (2017)	Yes	Yes	Yes	Yes
Muench et al. (2017)	Yes	Yes	Yes	Yes
Riordan et al. (2017)	Yes	Yes	Yes	No
Bock et al. (2016)	Yes	Yes	Yes	Yes
Andersson (2015)	Yes	Yes	Yes	Yes
Haug et al. (2015)	Yes	Yes	Yes	Yes
Riordan et al. (2015)	Yes	No	Yes	Can't tell
Suffoletto et al. (2015)<br>Suffoletto et al. (2014)*	Yes	Yes	Yes	No
Bendtsen & Bendtsen (2014)	Yes	Yes	Yes	No
Brendryen et al. (2014)	Yes	Yes	Yes	No
Gajecki et al. (2014)	Yes	Yes	Yes	No
Gustafson et al. (2014)	Yes	Yes	Yes	No
Lucht et al. (2014)	Yes	Yes	Yes	Yes
Mason et al. (2014)	Yes	No	Can't tell	Yes
Witkiewitz et al. (2014)	Yes	Yes	Yes	Yes
Agyapong et al. (2013)<br>Agyapong et al. (2012)*	Yes	Yes	Yes	Yes
Alessi & Petry (2013)	Yes	Yes	Yes	Yes
Hasin et al. (2013)	Yes	Yes	Yes	Yes
