# Supplementary material for: Mobile Health Interventions for Self-Control of Unhealthy Alcohol Use: Systematic Review
Source: JMIR Mhealth Uhealth. 2019 Jan 29;7(1):e10899. doi: 10.2196/10899 (PMC6371076; doi:10.2196/10899)
Supplement: Multimedia Appendix 5 [file mhealth_v7i1e10899_app5.pdf]

Appendix 5. Characteristics of the included studies

Author(s) (Year)	Country of origin	Sample size	Study arm
Aharonovich et al. (2017)	USA	47	HealthCall-S+MI (IG, n=23)<br>MI-only (CG, n=24)
Gajecki et al. (2017)	Sweden	186	Use TeleCoach Immediately after randomization (IG: n=93)<br>Use TeleCoach after 6 weeks/waitlist (CG: n=93)<br>Assessment-only control group (AG: n=144)
Muench et al. (2017)	USA	157	Gain-frame SMS (IG, n=31)<br>Loss-frame SMS (IG, n=31)<br>Statically Tailored SMS (IG, n=32)<br>Tailored Adaptive SMS (IG, n=33)<br>Assessment only SMS (CG, n=30)<br>Non-intervention (CG, n=19)
Riordan et al. (2017)	New Zealand	386	EMI+EMA (IG: n=59+135)<br>EMA only (CG: n=58+134)
Bock et al. (2016)	USA	60	Text Message Alcohol Program (IG, n=31)<br>General motivational SMS (CG, n=29)
Andersson (2015)	Sweden	1678	Single WEB (IG, n=323)<br>Repeated WEB (IG, n=318)<br>Single IVR (IG, n=329)<br>Repeated IVR (IG, n=334)<br>Screening only (CG, n=374)
Haug et al. (2015)	Switzerland	50	Aftercare programme SMS (IG, n=25)<br>Non-intervention (CG, n=25)
Riordan et al. (2015)	New Zealand	130	Intervention SMS (IG, n: NR)<br>Assessment only (CG, n: NR)
Suffoletto et al. (2015)<br>Suffoletto et al. (2014)*	USA	765	SMS + Feedback (IG, n=384)<br>Assessment only SMS (CG, n=196)<br>Non-intervention (CG, n=185)
Bendtsen & Bendtsen (2014)	Sweden	454	SMS (IG, n=229)<br>Email (CG, n=193)
Brendryen et al. (2014)	Norway	244	Brief + Intensive SMS (IG, n=125)<br>Brief + e-Booklet with non-behavior change of drinking (CG, n=119)
Gajecki et al. (2014)	Sweden	1932	PartyPlanner (IG, n=639)<br>Promillekoll (IG, n=643)<br>Non-intervention (CG, n=647)
Gustafson et al. (2014)	USA	349	A-CHESS (IG, n=170)<br>Non-intervention (CG, n=179)

Lucht et al. (2014)	Germany	80	SMS (IG: n=42)<br>TAU (CG: n=38)
Mason et al. (2014)	USA	18	MI + SMS (IG, n=8)<br>MI only (CG, n=10)
Witkiewitz et al. (2014)	USA	94	BASICS-Mobile (IG, n=32)<br>Mobile monitoring (CG, n=33)<br>Minimal assessment (CG, n=29)
Agyapong et al. (2013)<br>Agyapong et al. (2012)*	Ireland	54	Supportive SMS (IG, n=26)<br>Thanks SMS (CG, n=28)
Alessi & Petry (2013)	USA	30	Contingency management SMS (IG, n=15)<br>SMS (CG, n=15)
Hasin et al. (2013)	USA	258	MI+HealthCall (IG, n=88)<br>MI only (CG, n=82)<br>Advice/Education (CG, n=88)
AUD: alcohol use disorder<br>CG: control group<br>IG: intervention group<br>IVR: interactive voice response<br>MI: motivational interviewing<br>SMS: short message service<br>TAU: treatment as usual			
