# Supplementary material for: Mobile Health Interventions for Self-Control of Unhealthy Alcohol Use: Systematic Review
Source: JMIR Mhealth Uhealth. 2019 Jan 29;7(1):e10899. doi: 10.2196/10899 (PMC6371076; doi:10.2196/10899)
Supplement: Multimedia Appendix 6 [file mhealth_v7i1e10899_app6.pdf]

Appendix 6. Summary of included studies

Author(s) (Year) [Reference]	Context (patient characteristics; study setting; non-mobile co-intervention)	Theoretical base	Content	Delivery mode	Implementation procedure (length of intervention period; frequency; timing; baseline and follow-up assessment; other tricks)	Health outcome
Aharonovich et al. (2017)	AUD patients with HIV and use of drug, mean age: 51 years;<br>Community-based setting;<br>MI	NR (But the author claimed that the design of HealthCall app is theory-based)	(1) general queries about quantity of alcohol use, level of desire and commitment<br>(2) personalized alcohol use graph (3) reinforcement of alcohol abstinence<br>(4) daily "tip" video in which a counselor introduced skills to cut down alcohol use<br>(5) referrals to call counsellor<br>(6) reminders about sticking to the goal<br>(7) a reminder if not use the App more than 24 hours	App (HealthCall-S)	60 days;<br>Daily use (3mins/day);<br>Morning, afternoon or evening decided by the participants but need to be consistent every day;<br>Baseline assessment, 30-days assessment, 60-day assessment;<br>Provision of the study smartphone with the data plan, provision of $40 worth of gift card per assessment visit	No significant outcome for all following measurements: reduced DD, reduced DDD
Gajecki et al. (2017)	AUD students, mean age: 25 years;<br>University setting;<br>NR	NR	(1) queries about alcohol use<br>(2) a guideline for UAU<br>(3) prevention skills for dealing with relapse, including risk simulation analysis, alcohol refusal, relax, positive thinking exercises and urge surfing training	App (TeleCoachTM)	12 weeks;<br>Once weekly;<br>NR;<br>Baseline assessment, 6-week assessment, 12-week assessment;<br>NR	SD (6-week assessment): IG<CG (*P*=0.037), no longer significant at 12-week assessment<br>Frequency (6-week and 12-week assessments): IG<CG (*P*=0.041, *P*=0.034)<br>No significant outcome for all following measurements: binge occasions, average eBAC, peak eBAC
Muench et al. (2017)	Risky drinking adults, mean age: 43 years;<br>Community-based setting;<br>Participants learned NIAAA guidelines for safe alcohol use and a document regarding how to respond mobile assessment	Social learning theory, health belief model	(1) queries about alcohol use<br>(2a) loss-frame IG (LF): negative consequences of problem drinking<br>(2b) gain-frame IG (GF): benefits of reducing drinking to safe guidelines<br>(2c) statically tailored content IG (ST): tailored messages based on individual responses to baseline assessment<br>(2d) tailored adaptive IG (TA)<br>(2d1) individual tailored SMSs according to goal achievement<br>(2d2) 2 SMSs with participant's name at the heaviest typical drinking times<br>(2d3) a supportive message to response one of the automated system keywords sent by the participant	SMS	12 weeks;<br>Once weekly (4 SMSs) for content (1) and each SMS would be resent 3 more times in next 2 hours if no response within 1.5 hour for all IGs<br>Once daily for content (2a), (2b), and (2c) to LF, GF and ST&TA respectively at 6:00 pm<br>Once weekly for content (2d1) and (2d2);<br>Immediately for content (2d3);<br>Baseline assessment, 12-week assessment;<br>NR	Reduction of weekly SD: LF>MA (*P*=0.03), ST>MA (*P*=0.01), TA>MA (*P*=0.00)<br>Reduction of weekly HDD: LF>MA (*P*=0.05), ST>MA (*P*=0.01), TA>MA (*P*=0.00)<br>Increase of weekly abstinent days: GF>MA (*P*=0.03), ST>MA (*P*=0.04), TA>MA (*P*=0.02)
Riordan et al. (2017)	Risky drinking students, mean age: 18.5 years;	NR	Potential social consequence of UAU with colloquial tone	SMS	1 week (orientation week)<br>4 times week;	(1) In College 1:<br>SD during orientation week:

	University setting;<br>NR				Tuesday, Thursday, Friday and Saturday night at 19:00 and 21:00;<br>Baseline assessment, 1-week assessment, 1-semester assessment;<br>Provision of opportunity to win a mobile phone and prize of cash	IG<CG (*P*=0.18),<br>SD during the 1st semester: IG<CG (*P*=0.39)<br>(2) In college 2:<br>There are no significant differences in terms of alcohol use, but male students consumed significantly more alcohol than the female students (*P*<0.001).
Bock et al. (2016)	Risky drinking students, mean age: 22 years;<br>College setting;<br>NR	NR	(1) Alcohol facts<br>(2) strategies to limit alcohol use and alcohol-related risks<br>(3) motivation<br>(4) a supportive message to response one of the automated system keywords sent by the participant	SMS	6 weeks;<br>Once weekly (6 SMSs) for content (1)-(3), immediately for content (6);<br>Thursday and Sunday evening (1 SMS for each day), Friday and Saturday evening (2 SMSs for each day);<br>Baseline assessment; 6-week assessment; 12-week assessment;<br>NR	No significant outcome for all following measurements:<br>Drinking days per month<br>HDD per month<br>DDD<br>eBAC<br>Drinking days per past 2 weeks<br>Negative consequence<br>Strategies to limit drinking<br>Brief situational confidence
Andersson (2015)	Risky drinking students, mean age: 23 years;<br>University setting;<br>NR	NR	(1) personalized feedback for assessment results<br>(2) information on risk of negative consequences<br>(3) information on SD<br>(4) personalized recommendations for alcohol use<br>(5) information for increasing tolerance and goal-setting	IVR	1 week for single IG and 4 weeks for repeated IG;<br>Once daily;<br>NR;<br>Baseline assessment, 5-week assessment (4 weeks after intervention for single IG or 1 week after the intervention for repeat IG);<br>NR	Reduction of peak eBAC: IG (total)>CG (*P*=0.023), IG (repeated)>CG (*P*=0.046)<br>Reduction of AUDIT score: IG (total)>CG (*P*=0.000), IG (single)>CG (*P*=0.001), IG (repeated)>CG (*P*=0.001)<br>DDD, frequency of drinking, mean BAC: not significant
Haug et al. (2015)	AUD patients, mean age: 47 years;<br>Clinical setting;<br>NR	Theory of planned behaviour	(1) personalized queries for monitoring drinking goals<br>(2) personalized motivation of maintaining drinking goal according to their replies<br>(3) a reminder to the counsellor if no SMS reply over 2 days	SMS	6 months;<br>Once weekly (weeks 1–8) and bi-weekly (weeks 10-26) for content (1),<br>Immediately for content (2);<br>Monday at 18:00;<br>Baseline assessment; 6-month assessment<br>Phone calls from the therapist for support, empathy and further help	No significant outcome for AUDIT-C score
Riordan et al. (2015)	Risky drinking students, mean age: 22 years;<br>University setting;<br>NR	Social cognition models<br>Social cognitive theory	(1) social consequence of alcohol use<br>(2) health consequence of alcohol use<br>(3) queries about alcohol use	SMS	1 week (orientation week);<br>Once daily;	Reduction of SD during Orientation Week (women only): IG<CG (*P*<0.05)<br>Reduction of SD during the

		Theory of planned behavior<br>Self-determination theory<br>Model of action phases			Tuesday, Thursday and Saturday night (3 SMSs) at 19:30 for content (1)<br>Monday, Wednesday and Friday night (3 SMSs) at 19:30 for content (2)<br>Thursday, Friday, Saturday and Sunday (4 SMSs) for content (3);<br>Baseline assessment; 1-week assessment, 1-semester assessment;<br>NR	first semester (women only): IG<CG (*P*<0.05)
Suffoletto et al. (2015)<br>Suffoletto et al. (2014)*	Risky drinking emergency department patients, mean age: 22 years;<br>Hospital setting;<br>MI	Health belief model, information motivation behaviour model, theory of reasoned action, theory of planned behavior	(1) queries for reporting weekend drinking and binge plans<br>(2) decision not to set a low-risk goal<br>(2a) If "Yes", queries for reporting low-risk goal setting and feedback to promote reflection on drinking plan<br>(2b) If "No", feedback to strengthen low-risk drinking plan/goal<br>(3) queries for reporting alcohol use<br>(3a) feedback to support low-risk drinking behavior for adherence<br>(3b) feedback to promote reflection on alcohol consumption for non-adherence	SMS	12 weeks;<br>Twice weekly;<br>Thursday for contents (1) and (2), Sunday for contents (3);<br>Baseline assessment, 3-month assessment, 6-month assessment, 9-month assessment;<br>NR	HDD (at all assessments): IG<CG (significant intervention by time interaction)<br>DDD (at all assessments): IG<CG (significant intervention by time interaction)<br>Binge drinking prevalence (at all assessment): IG<CG (significant intervention by time interaction)<br>Alcohol-related injury prevalence (at 9-month assessment): IG<CG, not significant at others
Bendtsen & Bendtsen (2014)	Risky drinking students, mean age: 24 years;<br>University setting;<br>NR	Social cognition models, social cognitive theory, theory of planned behaviour, self-determination theory, model of action phases	(1) food for thought queries<br>(2) task<br>(3) challenges<br>(4) reflective	SMS	4 weeks;<br>4 times weekly (4 SMSs);<br>Wednesday for content (1),<br>Friday for content (2),<br>Saturday for content (3) and Sunday for content (4);<br>Baseline assessment, 4-week assessment;	No significant outcome for all following measurements:<br>Perceived drinking compared with peers<br>Motivation to change
Brendryen et al. (2014)	Risky drinking adults, mean age: 39 years;<br>Community-based setting;<br>NR	NR	(1) personalized comparison of the reported drinking habits to the recommended gender-matched low-risk drinking guidelines and national gender-matched averages<br>(2) 62 online sessions include four aspects: goal-setting and daily alcohol use track record, relapse prevention, emotion regulation and alcohol education.	SMS	6 months;<br>1 session daily for the first 56 sessions, 1 session weekly for sessions 57-60, 1 session monthly for sessions 61 and 62;<br>Available on demand;<br>Baseline assessment, 2-month assessment, 6-month assessment;	Weekly alcohol consumption: IG<CG (*P*=0.049)<br>FAST score: not significant

					NR	
Gajecki et al. (2014)	Risky drinking and AUD students, mean age: 25 years;<br>University setting<br>NR	Theory of planned behavior	(1) register for alcohol use<br>(2) eBAC result<br>(3) notification<br>(4) strategies to control alcohol use if a participant's alcohol use could lead to BAC>0.06%	App (Promillekoll)	7 weeks;<br>Real-time use;<br>Baseline assessment, 7-weeks assessment;<br>NR	SD per week, binge occasions, eBAC per week, peak eBAC per month: not significant<br>Drinking frequency (time-by-group interaction): IG>CG (*P*=0.001)<br>Drinking frequency (male): IG>CG (P=0.001)
		Theory of planned behavior	(1) a simulation of a planned drinking occasion for setting personal eBAC<br>(2) register for alcohol use<br>(3) eBAC result<br>(4) comparison of planned eBAC with actual eBAC	App (PartyPlanner)		No significant outcome for all the above measurements
Gustafson et al. (2014)	AUD patients, mean age: 38 years;<br>Nonprofit treatment organization setting<br>NR	Self-determination theory	(1) anonymous discussion<br>(2) question and answer<br>(3) instant library<br>(4) experience and knowledge sharing<br>(5) web-links<br>(6) distress easing<br>(7) warning when near high-risk alcohol place based on GPS tech<br>(8) brief survey to obtain patient data on negative affect, lifestyle balance, and recent substance use	App (A-CHESS)	8 months;<br>Real-time use, once weekly for content (8);<br>Baseline assessment, 4-month assessment, 8-month assessment, 12-month assessment;<br>NR	HDD (4-month and 12-month assessments): IG>CG (*P*=0.002, *P*=0.003), not significant at 8-month assessment<br>Prevalence and odds of abstinence (8-month and 12-month assessments, overall): IG>CG (*P*=0.004, *P*=0.002, *P*=0.003), not significant at 4-month assessment
Lucht et al. (2014)	AUD patients, mean age: 46 years<br>Hospital setting<br>NR	NR	(1) queries about alcohol use<br>(2) referrals for further help<br>(3) general automatic supportive feedback<br>(4) a reminder to the therapist if no SMS reply more than 24 hours	SMS	8 weeks;<br>Twice weekly;<br>Monday and Thursday;<br>Baseline assessment, 30-day assessment, 60-day assessment;<br>Phone calls from the therapist for support, empathy and further help	DD: No significant outcome<br>DDD: No significant outcome, but small effect in favor of IG<br>HDD: No significant outcome
Mason et al. (2014)	Risky drinking students, mean age: 19 years<br>College setting;<br>MI	Social network counselling	(1) queries about drinking facts<br>(2) social norms<br>(3) social risk<br>(4) protective behavioral "boosts" if requested	SMS	4 consecutive days;<br>4-6 SMSs daily;<br>NR;<br>Baseline assessment, 1-month assessment;<br>NR	Readiness to change alcohol use: IG↑, CG↓(*P*<0.01)<br>AUDIT, SD, Alcohol expectations, Importance of changing, Confidence in ability to change, intensions to reduce alcohol use, taking steps to reduce alcohol use: not significant
Witkiewitz et al. (2014)	Risky drinking students with smoking, mean age: 20.5 years<br>College setting;<br>MI	Cognitive-behavioral treatment	(1) normative feedback<br>(2) general or health information on alcohol use<br>(3) protective behavioral strategies for alcohol use<br>(4) alternative activities to alcohol use<br>(5) urge-surfing	App (BASICS-Mobile)	14 days;<br>Real-time use;<br>NR;<br>Baseline assessment, 14-day assessment, 1.5-month assessment;<br>NR	No significant outcome for all following measurements:<br>DDD<br>HDD<br>Young Adult Alcohol Problem Screening Test

			(6) decisional balance for alcohol use			
Agyapong et al. (2013)<br>Agyapong et al. (2012)*	AUD patients with unipolar depression, mean age: 48 years;<br>Hospital setting<br>NR	NR	(1) promoting alcohol abstinence<br>(2) dealing with cravings<br>(3) promoting medication adherence<br>(4) providing general support	SMS	3 months;<br>Twice daily (180 SMSs randomly sent);<br>10:00 and 19:00;<br>Baseline assessment, 3-month assessment, 6-month assessment (published in 2013);<br>NR	Cumulative abstinence duration (3-month and 6-month assessment): IG>CG (not significant);<br>Alcohol Abstinence Self Efficacy Scale (3-month assessment): IG>CG (P=0.02), no longer significance at 6-month assessment;<br>Days to first drink (6-month assessment): IG>CG (P=0.01), not significant at 3-month assessment;<br>DDD (3-month and 6-month assessment): IG<CG (not significant)
Alessi & Petry (2013)	Risky drinking adults, mean age: 34 years;<br>Community-based setting;<br>NR	Contingency management	(1) Reminder about BrAC video submission<br>(2) Reminder about possible compensation for valid on-time video	SMS	4 weeks;<br>1-3 times daily;<br>From 8 am to 11 pm (concentrated in evenings from 6 pm to 11 pm and weekends);<br>Baseline assessment, 4-weeks assessment;<br>Monetary incentives	Percentage of n-BrAC: IG>CG (*P*=0.00)<br>Longest abstinent days: IG>CG (*P*=0.00)<br>Reduction of DD: IG>CG (*P*=0.00)<br>Reduction of Alcohol Addiction Severity Index: IG>CG (*P*=0.01)<br>Reduction of overall Drinker Inventory of Consequences: IG>CG (*P*=0.00)<br>Reduction of DDD: not significant
Hasin et al. (2013)	HIV patients with risky drinking and AUD, mean age: 46 years<br>Clinical settings;<br>MI and training	NR	(1) queries about alcohol consumption<br>(2) queries about reasons for drinking/abstinence<br>(3) queries about mood<br>(4) queries about medication adherence<br>(5) queries about wellbeing<br>(6) reminder about continuous participation if missed two consecutive calls	IVR	60 days (first session for 30 days repeated second session for 30 days);<br>1-3 minutes daily;<br>Baseline assessment, 30-day assessment, 60-day assessment, 90-day assessment, 180-day assessment, 365-day assessment;<br>NR	Reduction of DD (60-day assessment): IG>CG ($P<0.01$);<br>IG>CG (*P*=0.03), no longer significance after 60-days

AUD: alcohol use disorder
CG: control group
DD: drinking day
DDD: drink per drinking day
HDD: heavy drinking day
IG: intervention group
IVR: interactive voice response
MI: motivational interviewing
NR: not reported
SMS: short message service
